# Enzyme-less nanopore detection of post-translational modifications within long polypeptides

**DOI:** 10.1038/s41565-023-01462-8

**Published:** 2023-07-27

**Authors:** Pablo Martin-Baniandres, Wei-Hsuan Lan, Stephanie Board, Mercedes Romero-Ruiz, Sergi Garcia-Manyes, Yujia Qing, Hagan Bayley

**Affiliations:** 1 Department of Chemistry, University of Oxford, Oxford, UK; 2 Department of Physics, Randall Centre for Cell and Molecular Biophysics and London Centre for Nanotechnology, King’s College London, London, UK; 3 Single Molecule Mechanobiology Laboratory, The Francis Crick Institute, London, UK

**Keywords:** Nanopores, Nanopores, Chemistry

## Abstract

Means to analyse cellular proteins and their millions of variants at the single-molecule level would uncover substantial information previously unknown to biology. Nanopore technology, which underpins long-read DNA and RNA sequencing, holds potential for full-length proteoform identification. We use electro-osmosis in an engineered charge-selective nanopore for the non-enzymatic capture, unfolding and translocation of individual polypeptides of more than 1,200 residues. Unlabelled thioredoxin polyproteins undergo transport through the nanopore, with directional co-translocational unfolding occurring unit by unit from either the C or N terminus. Chaotropic reagents at non-denaturing concentrations accelerate the analysis. By monitoring the ionic current flowing through the nanopore, we locate post-translational modifications deep within the polypeptide chains, laying the groundwork for compiling inventories of the proteoforms in cells and tissues.

## Abstract (web summary)

Electro-osmosis in an anion-selective α-hemolysin nanopore is used to capture, unfold and transport polypeptides of over 1,200 residues, which allows the mapping of post-translational modifications in polypeptide chains by monitoring the ionic current at a single-molecule resolution.

## Main

Single-molecule nanopore proteomics is gaining momentum^1^. Nanopore sequencing of ultralong DNA and RNA has enabled applications in basic science and medicine that challenge short-read technologies^2^. Modulation of the ionic current passing through a nanopore might also be used to distinguish and count the millions of proteoforms expressed from the 20,000 or so protein-encoding human genes. In this way, inventories would be obtained of variations such as post-translational modifications (PTMs) and the outcome of alternative RNA splicing, which are often present at multiple locations throughout a polypeptide chain^3^. Although folded proteins have been translocated through solid-state nanopores^4,5^ or protein nanopores of large sizes^6,7^, this approach has yet to be shown to locate modifications within a polypeptide sequence. Recently, PTMs have been detected within short peptides^8–10^. However, single-molecule proteoform identification requires the knowledge of the architecture of long polypeptide chains. Obtaining such information encounters two main roadblocks. First, proteins must be linearized for sequential PTM readout during nanopore translocation. Second, unlike DNA or RNA, polypeptides have a low-density and heterogeneous distribution of charge along their chains, which renders electrophoresis inapplicable as a means of translocation. One solution is to incorporate charged leader sequences, such as a single-stranded DNA^11–13^, to implement the electrophoretic capture and unfolding of proteins. However, as soon as the leader sequence exits the pore, a directional force is no longer present, and if the remaining polypeptide unfolds, it will diffuse through the pore in a partially extended conformation^11–13^. In another approach, unfoldases (for example, ClpX (refs. ^14,15^) or VATΔN (ref. ^16^)) have been employed to drive the translocation of tagged polypeptides through nanopores after electrophoretic threading. However, unlike the ratcheting enzymes used for DNA and RNA nanopore sequencing^17–19^, these unfoldases are not capable of the residue-by-residue translocation of polypeptides. Further, whether large PTMs will be tolerated during enzymatic translocation is unclear. Therefore, we aimed to establish a general non-enzymatic means to map modifications within full-length polypeptide chains and eventually to inventory the collection of proteoforms in individual cells rather than perform an ensemble analysis of peptide fragments.

Previously, we distinguished C-terminal PTMs (phosphorylated serines) in a model protein using the DNA leader approach^12^. However, the detection by nanopores of PTMs deep within a long polypeptide chain has remained a challenge. As an alternative to electrophoresis, electro-osmotic flow (EOF) within a charge-selective protein nanopore has been shown to modulate the binding and dissociation of neutral small molecules^20^, and promote the trapping of short peptides or folded proteins^21,22^. Recently, the unidirectional translocation of polypeptides through the wild-type (WT) staphylococcal α-hemolysin (αHL) pore was reported in the presence of a high concentration of guanidinium chloride (GdnHCl) (ref. ^23^). Computational analysis suggested that guanidinium cations line the interior of the pore and therefore alter the charge selectivity of the pore, generating EOF^23^. Here we use strong electro-osmosis directly attributable to the charged side chains in an engineered αHL pore to capture long underivatized polypeptides and detect modifications within them as they are unfolded and translocated. In addition, to aid in the co-translocational unfolding of protein analytes, chaotropic reagents (for example, GdnHCl and urea) are employed, which might contribute to the EOF through the pore.

## Electro-osmotic translocation of protein concatemers

**Fig. 1:**
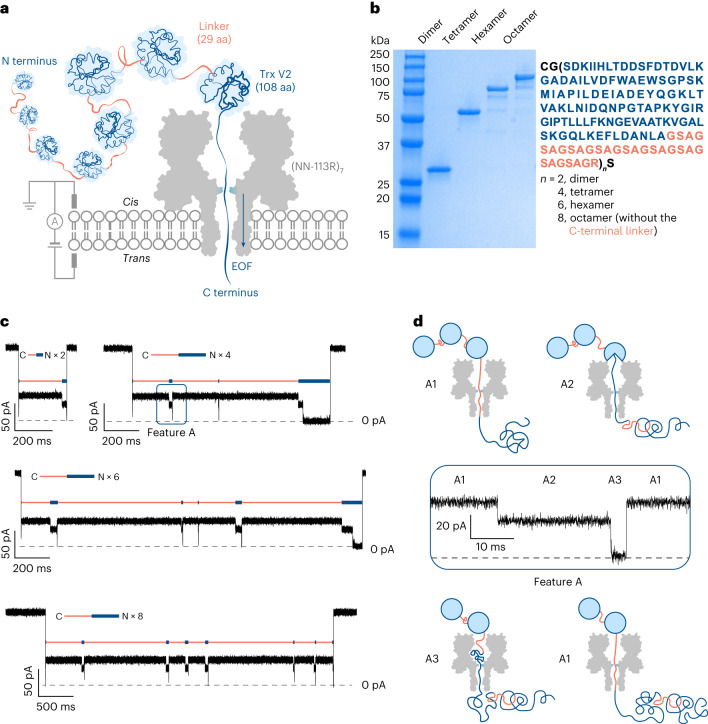
Electro-osmosis-driven translocation of Trx-linker concatemers through a protein nanopore. **a**, EOF in a charge-selective αHL nanopore (NN-113R)_7_ drives the sequential co-translocational unfolding of Trx units within a polyprotein of >1,000 aa. **b**, A sodium dodecyl sulfate–polyacrylamide gel showing the Trx-linker dimer (28 kDa), tetramer (55 kDa), hexamer (83 kDa) and octamer (110 kDa). **c**, Current recordings for the C-terminus-first translocation of a dimer, a tetramer, a hexamer and an octamer without post-acquisition filtering. The repeating features A are indicated by orange and blue bars. **d**. Zoomed-in view of the repeating feature A boxed in blue in **c** without post-acquisition filtering. Three levels are assigned as follows: A1, a linker within the pore; A2 and A3, different segments of partly unfolded Trx within the pore. Conditions in **c** and **d** are as follows: 750 mM GdnHCl, 10 mM HEPES, 5 mM TCEP at pH 7.2, Trx-linker concatemers (*cis*) (dimer: 2.23 μM; tetramer: 0.63 μM; hexamer: 0.25 μM; octamer: 0.81 μM), +140 mV (*trans*), 24 ± 1 °C. Source data

We constructed dimers, tetramers, hexamers and octamers of thioredoxin (Trx) (Fig. 1a,b, Supplementary Table 1 and Supplementary Fig. 1). Trx (108 amino acids (aa)) had the two catalytic cysteines removed (Trx: C32S/C35S)^11^. The Trx monomers were connected by 29 aa linkers, capable of spanning the 10-nm-long lumen of the αHL nanopore when fully extended (0.35 nm per aa). An exception was that the Trx-linker octamer had no C-terminal linker (Supplementary Table 1). We used a previously characterized anion-selective αHL mutant (NN-113R)_7_ (P_Na+_/P_Cl−_ = 0.33 (ref. ^20^)) to generate electro-osmosis. All the four Trx-linker concatemers were captured by (NN-113R)_7_ in the presence of 750 mM GdnHCl (Fig. 1c) at a capture rate ~25 times faster than that of the WT αHL pore (P_Na+_/P_Cl−_ = 0.78 (ref. ^20^)) (*k*_(octamer)_ ≈ 2.50 s^−1^ μM^−1^ with (NN-113R)_7_ versus ~0.11 s^−1^ μM^−1^ with (WT)_7_). The recording conditions were 750 mM GdnHCl, 10 mM HEPES at pH 7.2, +140 mV (*trans*), 24 ± 1 °C.

**Extended Data Fig. 1:**
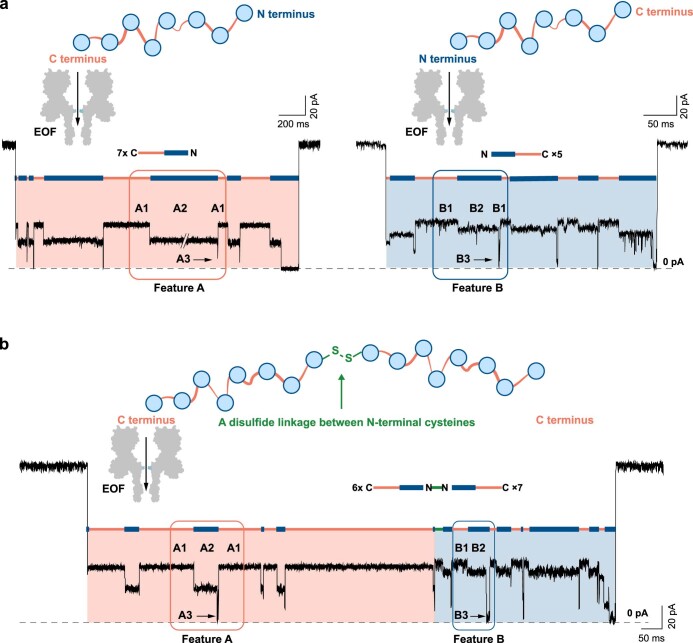
Repeating current features recorded during electroosmosis-driven concatemer translocation through a nanopore. **a**, Two repeating current features, A or B, were recorded with a charge-selective nanopore ((NN-113R)_7_) and Trx-linker octamers pre-treated with 5 mM tris(2-carboxyethyl)phosphine (TCEP) for 10 min before their addition to the cis compartment of the recording chamber. Conditions: 750 mM GdnHCl, 10 mM HEPES, 5 mM TCEP, pH 7.2, 0.81 μM Trx-linker octamer (cis), +140 mV (trans), 24 ± 1 °C. **b**, Without the TCEP pre-treatment, features A were always seen before features B when they occurred together within a single translocation event. The first two levels (B1 and B2) in features B have larger noise and higher I_res%_ compared with A1 and A2 recorded within a single polypeptide translocation event by the same pore (A1: I_res%_= 35 ± 1 %, I_r.m.s._ = 1.1 ± 0.1 pA, N = 25; A2: I_res%_ = 21 ± 1%, I_r.m.s._ = 1.5 ± 0.2 pA, N = 25; B1: I_res%_= 38 ± 1 %, I_r.m.s._ = 1.7 ± 0.4 pA, N = 39; B2: I_res%_ = 32 ± 1%, I_r.m.s._ = 2.0 ± 0.5 pA, N = 39; I_r.m.s._ values for each level were reported without subtraction of the noise of the pore; number of individual levels from multiple polypeptide translocation events recorded by the same pore are specified). The translocating molecules, which gave sequential A and B features, were assigned as dimers of octamers linked by a disulfide bond between the two N-terminal cysteines. Therefore, in the unlinked molecules (see ‘**a**’), C terminus-first translocation occurred when features A were observed and N terminus-first translocation occurred when features B were observed. The recorded repeating features are indicated by orange and blue bars. Conditions: 750 mM GdnHCl, 10 mM HEPES, pH 7.2, 0.81 μM Trx-linker octamer (cis), +140 mV (trans), 24 ± 1 °C. All traces were filtered at 2 kHz for clarity; transient A3 levels were truncated by filtering and therefore deviated from ~0 pA.

Electro-osmosis-driven concatemer translocation produced current patterns containing repeating features (Fig. 1c and Extended Data Fig. 1). The most abundant feature, A, consisted of three levels (A1, A2 and A3) (Fig. 1c,d). The percentage residual current (*I*_res%_) for each level in feature A was consistent across all such events for each polypeptide translocation and between all the individual concatemers observed with the same or different pores (Supplementary Table 2). A spike to ~0 pA was seen at the beginning of almost all the translocation events and was speculated to represent the rapid unfolding and translocation of the first Trx-linker unit. The spike was followed by up to *n* – 1 repeats of the three-step feature A (*n* is the number of Trx-linker units in the concatemer), which unambiguously demonstrated the stepwise translocation of entire polypeptide chains one unit at a time.

Less often, a different repeating element B was recorded (Extended Data Fig. 1a and Supplementary Table 3). Further, when two identical concatemers were linked by a disulfide bond between the N-terminal cysteines, feature B occurred only after feature A within each translocation event (Extended Data Fig. 1b). Therefore, we assigned these two features as C-terminus-first (A) and N-terminus-first (B) translocation events. In the presence of a C-terminal linker (for example, Trx tetramer; Fig. 1b), the ratio of C-terminus-first versus N-terminus-first translocation events was ~2:1 at +140 mV (Supplementary Table 3); in the absence of the C-terminal leader sequence (for example, Trx octamer; Fig. 1b), the C-terminus-first pattern dominated (C versus N = ~10:1 at +140 mV) (Supplementary Table 3).

The repeating feature A was lost at a GdnHCl concentration of 3 M (Supplementary Fig. 2). At 750 mM GdnHCl, ~12% of the translocated octamers produced a maximum of seven repeats of feature A following the initial spike (Supplementary Table 4); a kinetic analysis revealed two populations of A3: one had a mean dwell time ~500 times longer than the other at +140 mV (<*τ*_A3_> = 320 ± 60 ms versus 0.69 ± 0.04 ms) (Supplementary Table 5). The longer-lived A3 (*τ*_A3_ > 10 ms) was seen in 25% of the final features A recorded as the translocation of an octamer was completed, but only in 3% of the preceding features A. Tentatively, we assign level A1 as a threaded linker preceding the C terminus of a folded Trx unit; level A2 as the C-terminal portion of a partially unfolded Trx unit extended into the nanopore; and level A3 as the spontaneous unfolding and passage of the remaining Trx polypeptide through the nanopore (Fig. 1d). The dominant absence of a multilevel feature for the first unit and an extended duration for the last unit suggest that the unfolding kinetics of Trx units differ when the polypeptide chain is unable to fully span the lumen of the nanopore.

**Fig. 2:**
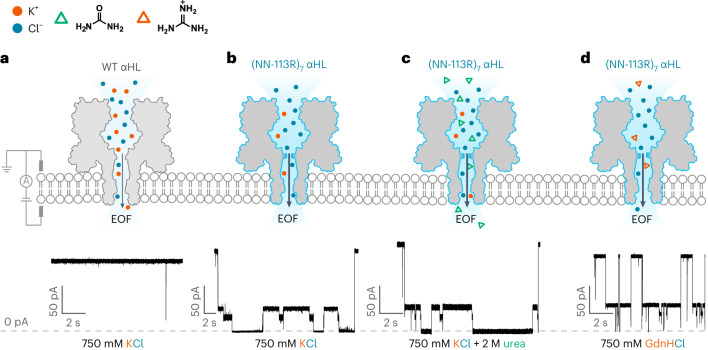
Chaotrope-facilitated electro-osmotic translocation of the Trx-linker octamers through a nanopore. **a**, Translocation of Trx-linker octamers through a weakly anion-selective WT αHL was not observed in the absence of a chaotrope. **b**–**d**, Current traces showing the translocation of Trx-linker octamers through the electro-osmotically active nanopore (NN-113R)_7_ in the presence of 750 mM KCl (**b**), 750 mM KCl and 2 M urea (**c**) or 750 mM GdnHCl (**d**) with 2 kHz post-acquisition filtering. The use of non-denaturing concentrations of chaotropic agents (urea and GdnHCl) accelerated the co-translocational unfolding of the Trx units. Conditions: 10 mM HEPES at pH 7.2, 0.81 μM Trx-linker octamer (*cis*), +140 mV (*trans*), 24 ± 1 °C with 750 mM KCl (**a** and **b**); 2 M urea and 750 mM KCl (**c**); 750 mM GdnHCl (**d**).

Similar repeating features were also seen in the absence of GdnHCl (recording conditions: 750 mM KCl, 10 mM HEPES at pH 7.2, +140 mV (*trans*), 24 ± 1 °C) or in the presence of a non-denaturing concentration of urea (recording conditions: 2 M urea, 750 mM KCl, 10 mM HEPES at pH 7.2, +140 mV (*trans*), 24 ± 1 °C). Under these conditions, the translocation kinetics were slower (Fig. 2), and these options were not pursued further.

## Detection of PTMs during electro-osmotic translocation

**Fig. 3:**
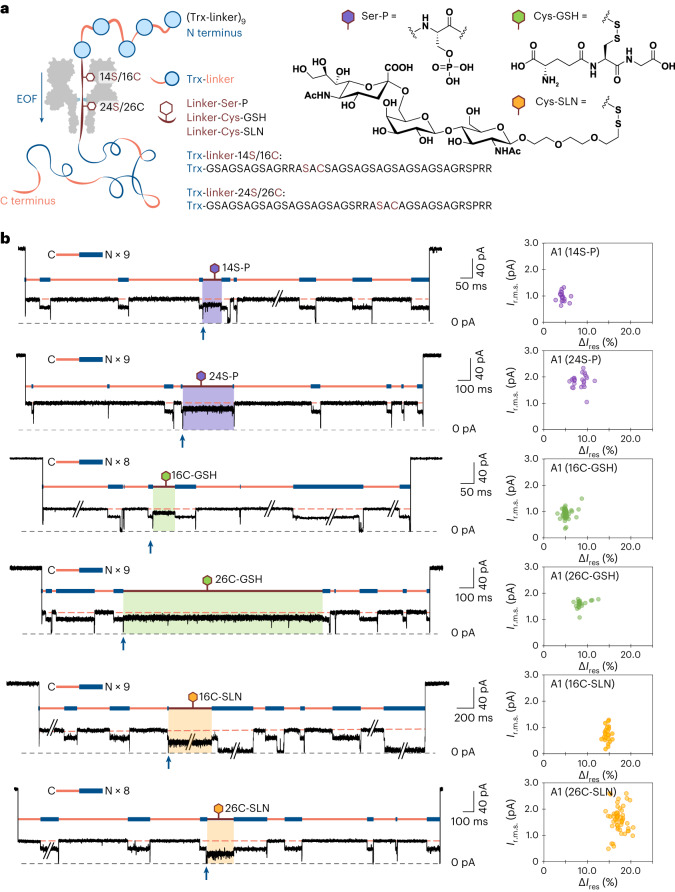
Detection of PTMs in protein concatemers traversing a nanopore driven by EOF. **a**, Trx-linker nonamers tested with a charge-selective nanopore ((NN-113R)_7_) containing an RRASAC sequence within the central linker, which was post-translationally phosphorylated (purple), S-glutathionylated (green) or glycosylated (yellow). **b**, Recordings of C-terminus-first translocation events of Trx-linker nonamers (left), showing a distinct level A1 (boxed in purple, green or yellow) in the presence of a PTM compared with the level A1 of unmodified units (orange dash). Traces have been filtered at 2 kHz; transient A3 levels were truncated by filtering and therefore deviate from ~0 pA. The A3 level produced by the translocation of an unmodified unit before the modified linker is indicated with a blue arrow and each of the features A is indicated by orange and blue bars. The number of repeats of feature A within the polypeptide translocation event shown is specified. Scatter plots of *I*_r.m.s._ and Δ*I*_res%_ for individual polypeptide translocation events (right), where Δ*I*_res%_ = <*I*_res%_(A1, Trx-linker)> – *I*_res%_(A1, Trx-linker + PTM), where <*I*_res%_(A1, Trx-linker)> is the mean *I*_res%_ value of the remaining A1 levels for unmodified repeat units within an individual translocation event. Conditions, 375 mM GdnHCl, 375 mM KCl, 10 mM HEPES at pH 7.2, 1.2 μM Trx-linker nonamer (*cis*), +140 mV (*trans*), 24 ± 1 °C. Source data

To determine whether PTMs near the middle of a long polypeptide chain could be located during electro-osmosis-driven translocation, we constructed Trx-linker nonamers containing a modification site (RRASAC) at two different positions in the central linker (Supplementary Table 1 and Supplementary Fig. 3) for serine phosphorylation (14S-P or 24S-P) or cysteine-directed glutathionylation or glycosylation (16C-GSH, 26C-GSH, 16C-SLN or 26C-SLN) (Fig. 3a). In the presence of a phosphate group (P) or glutathione (GSH) or 6′-sialyllactosamine (SLN), level A1 for the modified units exhibited a smaller *I*_res__%_ value and higher root-mean-square noise (*I*_r.m.s._) than that of the unmodified segments within an individual polypeptide (Fig. 3b and Supplementary Table 6). Furthermore, the average increment in the current blockade was roughly proportional to the mass of the PTM, with the phosphate giving the smallest increment and the trisaccharide giving the largest (Supplementary Table 6); however, there was substantial overlap between the 14S-P/24S-P and 16C-GSH/26C-GSH populations (Fig. 3b and Supplementary Fig. 4). Mixed nonamers containing different PTMs at the same site (26C-GSH and 26C-SLN) were discriminated by the same pore (Supplementary Fig. 5). All the three PTMs tested caused a smaller current blockade at serine 14 (14S) or cysteine 16 (16C) than at serine 24 (24S) or cysteine 26 (26C) (Fig. 3b). Given that 14S/16C must be closer to the *cis* opening of the αHL pore than 24S/26C in a C-terminus-first threading configuration, it is probable that the central constriction of the pore is located closer to 24S/26C (Fig. 3a and Supplementary Fig. 6). The findings also suggest that the polypeptide might not be fully extended under the EOF (Supplementary Fig. 6 shows further analysis), which corroborates the force spectroscopy data for polypeptides under forces of <20 pN (refs. ^24,25^).

## Conclusions

Here we have established that electro-osmotically active nanopores can capture and unfold individual proteins comprising long (>1,200 aa) polypeptide chains for PTM identification and localization. To a first approximation, the electro-osmotic force acting on a polypeptide remains constant during translocation, creating a unidirectional bias desirable for placing PTMs in sequence. In contrast, the overall time for unforced polypeptide translocation scales roughly as the square of its length, because the polypeptide chain can move back and forth before diffusing out of the pore^26^. This is the case within an electro-osmotically inactive nanopore after the exit of a charged leader sequence^11^ or immediately after a protein domain has unfolded during movement propelled by a motor protein^14^, which is not ideal for the sequential detection of modification sites within individual polypeptide chains. Furthermore, as a label-free method, our approach circumvents the need to derivatize proteins at either the N or C terminus for electrophoretic translocation, which could be problematic for eukaryotic proteins due to the widespread presence of N-acetylation and the lack of efficient N- or C-terminus-specific chemistries.

Here we have located PTMs in linkers deep within long polyprotein chains by exploiting stepwise unfolding. Our encouraging results lay the groundwork for building inventories of underivatized full-length proteoforms from cells and tissues. The detection of PTMs in freely translocating domains require the slowing and stretching of polypeptide chains, which might be produced by physical effects (for example, heat and voltage ramps), nanopores with different internal geometries and surface charge and the use of weakly bound ligands. Recording at megahertz acquisition rates^27^ might also prove advantageous. These endeavours are beyond the scope of the present work and indeed depend on the advances we report here.

Our strategy will be readily transferable to nanopore sequencing devices (for example, the MinION) for highly parallel PTM profiling, which will be useful for producing inventories of full-length human proteoforms, which are ~500 aa in median length^28^. To achieve the complete characterization of the proteoforms in individual cells, our approach faces challenges. For example, because proteins vary in their resistance to unfolding, it will be problematic to establish universal conditions for the translocation of all the protein components of a cell. Similarly, the capture rate will probably differ between proteins. To this end, voltage sweeps might be used in combination with denaturants to promote protein capture and facilitate co-translocational unfolding. Further, compact PTMs (for example, methylation) might be challenging to directly detect. Ligand-assisted detection might be performed with antibodies or chemical binders. In summary, our enzyme-less approach, targeting full-length proteins, presents a viable nanopore technology, ultimately allowing comprehensive proteoform inventories to be established for tissues and single cells. These massive sets of information will extend beyond what is recognized from DNA and RNA sequencing and potentially unveil yet-unknown aspects of the biology of cells and tissues.

## Methods

### Construction of Trx-linker concatemer genes

All the reagents were purchased from New England Biolabs (NEB) and DNA oligonucleotides were obtained from Integrated DNA Technologies, unless otherwise indicated. Trx-linker concatemer genes were prepared as previously described^29^. Briefly, the Trx-linker monomer gene was amplified with a 5′ primer containing a BamHI restriction site and a 3′ primer containing a BglII restriction site, which permitted the in-frame cloning of the monomer into the vector pQE30 (QIAGEN). Synthetic genes encoding the concatemers were then constructed by the iterative cloning of monomer into monomer, dimer into dimer and tetramer into tetramer. To aid purification, an N-terminal SUMO tag was inserted between the His6 tag and the first monomer unit. In addition, N-terminal cysteine-glycine codons were included to give the final concatemer constructs: His6-SUMO-CysGly-(Trx-linker)_*n*_ (*n* = 2, 4, 6) and His6-SUMO-CysGly-(Trx-linker)_7_Trx.

To produce Trx-linker nonamers (His6-SUMO-(Trx-linker)_*n*_, *n* = 9) containing a modification site, the N-terminal cysteine-glycine codons were removed from the tetramer gene and a DNA cassette was designed to contain two terminal restriction sites (BamHI and BglII) and two internal restriction sites (KpnI and AvrII) (5′-p GATCCGGTACCGGCGGTCCTAGG AGATCTGGCGGTA-3′ and 5′-p GCCATGGCCGCCAGGATCCTCTAGACCGCCATTCGA-3′). Using the interactive cloning strategy described above, a ‘cloneable’ Trx-linker octamer gene was assembled with the DNA cassette as the middle unit flanked by two Trx-linker tetramer genes (that is, the final construct is His6-SUMO-(Trx-linker)_4_-KpnI-AvrII-(Trx-linker)_4_). A Trx-linker monomer mutant gene encoding an RRASAC peptide motif was created by site-directed insertion (forward primer, 5′- AGCGCCTGCGCGGGTTCTGCTGGTTCC-3′; reverse primer, 5′-CGCACGGCG GCTCCCTGCACTTCCGGC-3′) and subsequently cloned in between the KpnI and AvrII sites within the Trx-linker octamer to give (Trx-linker)_4_-Trx-linker(RRASAC)-(Trx-linker)_4_. The placement of a single correctly oriented insert was confirmed by sequencing using primers targeting the KpnI and AvrII ligation sites (forward primer, 5′-TGCGAGCGCCTGCGGTGG-3′; reverse primer, 5′-ACGCTCGCGGACGCCACC-3′).

### Expression and purification of Trx-linker concatemers

Genes encoding the N-terminal His6-SUMO-tagged concatemers of Trx were cloned into the pOP3SU plasmid (kindly provided by M. Hyvönen). BLR(DE3) competent cells (Novagen) were transformed with the plasmids and grown in a Luria broth medium supplemented with ampicillin (100 µg ml^–1^) at 37 °C with continuous shaking (250 r.p.m.). Protein expression was induced in the exponential growth phase (OD_600_ = 0.6) with isopropyl-β-d-1-thiogalactopyranoside (0.5 mM final concentration). After 8 h, the cells were harvested by centrifugation (10 min, 5,000×*g*), resuspended in a binding buffer (30 mM Tris HCl, 250 mM NaCl, 25 mM imidazole at pH 7.2) supplemented with a protease inhibitor cocktail (cOmplete, EDTA free; Roche) and lysed by sonication. Cell debris was removed by centrifugation at 20,000×*g* for 45 min, and the supernatant loaded onto a HisTrap HP column (5 ml, Cytiva) at 0.2 ml min^–1^. The column was washed with 50 ml of the binding buffer before single-step elution with 15 mL of 30 mM Tris HCl, 250 mM NaCl, 300 mM imidazole at pH 7.2. A single peak containing the almost pure protein was collected and dialysed (Slide-A-Lyzer G2 Dialysis Cassette, 10,000 molecular weight cutoff, 30 ml; Thermo Fisher) for 3 h against 4 l of dialysis buffer (50 mM Tris HCl, 250 mM NaCl, 2 mM 1,4-dithio-d-threitol (DTT) at pH 8.0), at 4 °C with continuous stirring, to remove excess imidazole. After injecting His6-tagged Ulp1 protease into the dialysis cassette at a molar concentration ratio of 1:200 (Ulp1:Trx-linker concatemer), the mixture was transferred into a fresh dialysis buffer overnight for SUMO-tag cleavage. The cassette was then transferred one last time into fresh dialysis buffer without DTT for 4 h. The dialysed protein was loaded onto a column packed with HisPur Ni-NTA Agarose Resin (5 ml, Thermo Fisher) equilibrated with a binding buffer (50 mM Tris HCl, 250 mM NaCl at pH 8.0) and the flow through was reapplied five more times. The final flow through containing the His6-SUMO-free protein was aliquoted and flash frozen for storage at −80 °C.

### Expression and purification of SUMO protease Ulp1

The Pfget19_Ulp1 plasmid (Addgene) containing a His6-tagged Ulp1 gene was transformed into T7 Express competent cells (NEB) and grown in a Luria broth medium supplemented with kanamycin (100 μg ml^–1^) at 37 °C with shaking (250 r.p.m.). Expression was induced at OD_600_ = 0.5 with isopropyl-β-d-1-thiogalactopyranoside (0.5 mM). Cells were harvested after 3 h by centrifugation, resuspended in lysis buffer (4 ml g^–1^; 50 mM Tris HCl, 300 mM NaCl, 10 mM imidazole at pH 7.5) supplemented with lysozyme (1 mg ml^–1^) and incubated on ice for 30 min before sonication. The lysate was spun at 20,000 r.p.m. for 45 min to remove the cell debris and the supernatant was applied to a column packed with HisPur Ni-NTA Agarose Resin (5 ml, Thermo Fisher) and equilibrated with a binding buffer (50 mM Tris HCl, 300 mM NaCl at pH 7.5). The column was washed with 10 column volumes of wash buffer (50 mM Tris HCl, 300 mM NaCl, 20 mM imidazole at pH 7.5) and the protein was eluted with 10 ml of elution buffer (50 mM Tris HCl, 300 mM NaCl, 300 mM imidazole at pH 7.5). The eluted protein was dialysed against a storage buffer (50 mM Tris HCl, 200 mM NaCl, 2 mM 2-mercaptoethanol) overnight, aliquoted and flash frozen as a 50% stock in glycerol.

### Phosphorylation of Trx-linker concatemers

Trx-linker concatemers (1 mg ml^–1^) were incubated with 50,000 units of the catalytic subunit of cAMP-dependent protein kinase (NEB)—which recognizes the RRAS motif within the central linker of the Trx-linker nonamer—in a protein kinase buffer (50.0 mM Tris HCl at pH 7.5,10.0 mM MgCl_2_, 0.1 mM EDTA, 4.0 mM DTT, 0.01% Brij 35 and 2.0 mM ATP) (NEB) at 30 °C for 1 h. The solution was then supplemented with additional ATP at a final concentration of 2 mM and DTT at a final concentration of 2 mM before overnight incubation at 30 °C. Trx-linker concatemers were purified and concentrated using centrifugal filters (Amicon Ultra 0.5 ml, 100 K), aliquoted and flash frozen for storage at −20 °C (10 mM HEPES at pH 7.2 and 750 mM KCl). Phosphorylation of the Trx-linker concatemers at a single site was verified by liquid chromatography–mass spectrometry.

### Modification of cysteines on Trx-linker concatemers

Reagents were purchased from Sigma-Aldrich, unless otherwise indicated. Trx-linker nonamer was first treated with tris(2-carboxyethyl)phosphine (TCEP) (70 to 100 eq) at 32 °C for 2 h in a protein storage buffer (50 mM Tris HCl, 250 mM NaCl at pH 8.0). Excess TCEP was removed by a desalting column (PD MiniTrap G-25 column, Cytiva). To glutathionylate the Trx-linker nonamer, the reduced protein was reacted with oxidized glutathione (100 eq) at 32 °C overnight in a protein storage buffer (50 mM Tris HCl, 250 mM NaCl at pH 8.0) before desalting to remove the excess reagent. The modified protein was aliquoted and flash frozen for storage at −20 °C. To glycosylate the Trx-linker nonamers, the reduced protein was reacted first with 2,2′-dithiodipyridine (20 eq) at 32 °C overnight in the protein storage buffer (50 mM Tris HCl, 250 mM NaCl at pH 8.0). After the removal of excess 2,2′-dithiodipyridine with a desalting column, the activated nonamer was reacted with the 6′-sialyllactosamine derivative (NeuAcα(2-6)LacNAc-PEG3-Thiol, 5 eq; Sussex Research Laboratories) overnight at 32 °C in a protein storage buffer (50 mM Tris HCl, 250 mM NaCl at pH 8.0). Modified nonamers were desalted (PD MiniTrap G-25 column, Cytiva), aliquoted and flash frozen for storage at −20 °C. The occurrence of glutathionylation or glycosylation at single sites was verified by liquid chromatography–mass spectrometry.

### Single-channel recording

Planar lipid bilayers of 1,2-diphytanoyl-*sn*-glycero-3-phosphocholine (Avanti Polar Lipids) were formed by using the Müller–Montal method on a 50-μm-diameter aperture made in a Teflon film (25 μm thick, Goodfellow) separating two 500 μl compartments (*cis* and *trans*) of the recording chamber. Each compartment was filled with a recording buffer (750 mM GdnHCl, 1.5 M GdnHCl, 3.0 M GdnHCl, 2.0 M urea/750 mM KCl or 750 mM KCl, 10 mM HEPES, 5 mM TCEP at pH 7.2 for Trx-linker dimer, tetramer, hexamer and octamer; 375 mM GdnHCl/375 mM KCl, 10 mM HEPES at pH 7.2 for Trx-linker nonamers). To record with Trx-linker dimer, tetramer, hexamer or octamer and ensure a reduced N-terminal cysteine, pretreatment of the protein samples with 5 mM TCEP was carried out for 10 min at room temperature. This pretreatment was not carried out with nonamers which lacked the N-terminal cysteine residue. Trx-linker concatemers were added to the *cis* compartment (dimer, 2.20 μM; tetramer, 0.63 μM; hexamer, 0.25 μM; octamer, 0.81 μM; nonamer, 1.20 μM). Ionic currents were measured at 24 ± 1 °C by using Ag/AgCl electrodes connected to the headstage of an Axopatch 200B amplifier. After a single (NN-113R)_7_ pore had inserted into the bilayer, the solution was replaced with a fresh buffer by manual pipetting, to prevent further insertions. Signals were low-pass filtered at 10 kHz and sampled at 50 kHz with a Digidata 1440A digitizer (Molecular Devices).

### Data analysis

To establish the current signatures for the stepwise co-translocational unfolding of Trx concatemers, current traces were analysed using Clampfit 10.7 (Molecular Devices). The remaining current as a percentage of the open-pore current (*I*_res%_) was calculated for each step in individual A or B features (for example, *I*_res%_(A1) = *I*_A1_/*I*_open_ × 100%). The standard deviations were derived from data for Trx-linker units collected using separate pores. Trx-linker units that produced a level A3 or B3 with a dwell time of <1 ms were excluded from the *I*_res%_ analysis due to possible undersampling. Root-mean-square noise values (*I*_r.m.s._) for each current level were measured from current traces after the application of a post-recording filter of 2 kHz. Unless otherwise stated, the noise of the open pore was subtracted as follows: *I*_r.m.s._^2^ = *I*_r.m.s._(A1)^2^ – *I*_r.m.s._(open pore)^2^. To obtain the stepwise kinetic profiles of the co-translocational unfolding of Trx concatemers, current traces were idealized using Clampfit 10.7. The dwell-time analysis was performed by using the maximum interval likelihood algorithm of QUB 2.0 software (https://qub.mandelics.com/).

## Online content

Any methods, additional references, Nature Portfolio reporting summaries, source data, extended data, supplementary information, acknowledgements, peer review information; details of author contributions and competing interests; and statements of data and code availability are available at 10.1038/s41565-023-01462-8.

### Supplementary information

Supplementary Information Supplementary Tables 1–6, Figs. 1–6 and References.

### Source data

Source Data Fig. 1 Unprocessed sodium dodecyl sulfate–polyacrylamide gel.

Source Data Fig. 3 Statistical source data.

## Data Availability

Source data are provided with this paper. All other data pertaining to this study are available from the corresponding authors upon reasonable request.
