# Supplementary material for: Enzyme-less nanopore detection of post-translational modifications within long polypeptides
Source: Nat Nanotechnol. 2023 Jul 27;18(11):1335–40. doi: 10.1038/s41565-023-01462-8 (PMC10656283; doi:10.1038/s41565-023-01462-8)
Supplement: Supplementary file 1 — Supplementary Tables 1–6, Figs. 1–6 and References. [file 41565_2023_1462_MOESM1_ESM.pdf]

nature nanotechnology

Article

https://doi.org/10.1038/s41565-023-01462-8

Enzyme-less nanopore detection of post-translational modifications within long polypeptides

In the format provided by the authors and unedited

Table of Contents:

Table S1 Sequences of the thioredoxin-linker concatemers 2
Table S2 Percentage residual currents ($I_{res\%}$) for the three levels of repeating feature A recorded during C-terminus first concatemer translocation 4
Table S3 Frequency of C terminus-first or N terminus-first translocation events recorded with Trx-linker concatemers 5
Table S4 Percentages of concatemer translocation events with N numbers of feature A repeats 6
Table S5 Mean dwell times (<τ>) for the three levels of repeating feature A (A1, A2, A3) recorded during the C-terminus first translocation of Trx-linker octamers through a single nanopore 7
Table S6 Percentage residual current ($I_{res\%}$) and root-mean-square noise (I_{RMS}) characteristics of individual modifications on Trx-linker nonamers 8
Fig. S1 LC-MS characterization of Trx-linker concatemers 9
Fig. S2 Electroosmosis-driven translocation of Trx-linker octamers through a nanopore 10
Fig. S3 LC-MS characterization of Trx-linker nonamers 11
Fig. S4 Identification and positional discrimination of PTMs in protein concatemers translocated by electroosmotic flow through a nanopore 12
Fig. S5 Identification of PTMs in a mixture of two concatemers 13
Fig. S6 Positions of modification sites during translocation through an αHL pore 14
References 15

Table S1 Sequences of the thioredoxin-linker concatemers

Dimer (Trx-linker)$_2$

CGSDKIIHLTDDSFDTDVLKADGAILVDFWAEWSGPSKMIAPILDEIADEYQGKLTVAKLNIDQNPGTAPKY
GIRGIPTLLLFKNGEVAATKVGALSKGQLKEFLDANLAGSAGSAGSAGSAGSAGSAGSAGSAGSAGRSDK
IIHLTDDSFDTDVLKADGAILVDFWAEWSGPSKMIAPILDEIADEYQGKLTVAKLNIDQNPGTAPKYGIRGIP
TLLLFKNGEVAATKVGALSKGQLKEFLDANLAGSAGSAGSAGSAGSAGSAGSAGSAGSAGRS

Tetramer (Trx-linker)$_4$

CGSDKIIHLTDDSFDTDVLKADGAILVDFWAEWSGPSKMIAPILDEIADEYQGKLTVAKLNIDQNPGTAPKY
GIRGIPTLLLFKNGEVAATKVGALSKGQLKEFLDANLAGSAGSAGSAGSAGSAGSAGSAGSAGSAGRSDK
IIHLTDDSFDTDVLKADGAILVDFWAEWSGPSKMIAPILDEIADEYQGKLTVAKLNIDQNPGTAPKYGIRGIP
TLLLFKNGEVAATKVGALSKGQLKEFLDANLAGSAGSAGSAGSAGSAGSAGSAGSAGSAGRSDKIIHLTD
DSFDTDVLKADGAILVDFWAEWSGPSKMIAPILDEIADEYQGKLTVAKLNIDQNPGTAPKYGIRGIPTLLLFK
NGEVAATKVGALSKGQLKEFLDANLAGSAGSAGSAGSAGSAGSAGSAGSAGSAGRSDKIIHLTDDSFDT
DVLKADGAILVDFWAEWSGPSKMIAPILDEIADEYQGKLTVAKLNIDQNPGTAPKYGIRGIPTLLLFKNGEVA
ATKVGALSKGQLKEFLDANLAGSAGSAGSAGSAGSAGSAGSAGSAGSAGRS

Hexamer (Trx-linker)$_6$

CGSDKIIHLTDDSFDTDVLKADGAILVDFWAEWSGPSKMIAPILDEIADEYQGKLTVAKLNIDQNPGTAPKY
GIRGIPTLLLFKNGEVAATKVGALSKGQLKEFLDANLAGSAGSAGSAGSAGSAGSAGSAGSAGSAGRSDK
IIHLTDDSFDTDVLKADGAILVDFWAEWSGPSKMIAPILDEIADEYQGKLTVAKLNIDQNPGTAPKYGIRGIP
TLLLFKNGEVAATKVGALSKGQLKEFLDANLAGSAGSAGSAGSAGSAGSAGSAGSAGSAGRSDKIIHLTD
DSFDTDVLKADGAILVDFWAEWSGPSKMIAPILDEIADEYQGKLTVAKLNIDQNPGTAPKYGIRGIPTLLLFK
NGEVAATKVGALSKGQLKEFLDANLAGSAGSAGSAGSAGSAGSAGSAGSAGSAGRSDKIIHLTDDSFDT
DVLKADGAILVDFWAEWSGPSKMIAPILDEIADEYQGKLTVAKLNIDQNPGTAPKYGIRGIPTLLLFKNGEVA
ATKVGALSKGQLKEFLDANLAGSAGSAGSAGSAGSAGSAGSAGSAGSAGRSDKIIHLTDDSFDTDVLKAD
GAILVDFWAEWSGPSKMIAPILDEIADEYQGKLTVAKLNIDQNPGTAPKYGIRGIPTLLLFKNGEVAATKVG
ALSKGQLKEFLDANLAGSAGSAGSAGSAGSAGSAGSAGSAGSAGRSDKIIHLTDDSFDTDVLKADGAILV
DFWAEWSGPSKMIAPILDEIADEYQGKLTVAKLNIDQNPGTAPKYGIRGIPTLLLFKNGEVAATKVGALSKG
QLKEFLDANLAGSAGSAGSAGSAGSAGSAGSAGSAGSAGRS

Octamer (Trx-linker)$_8$

CGSDKIIHLTDDSFDTDVLKADGAILVDFWAEWSGPSKMIAPILDEIADEYQGKLTVAKLNIDQNPGTAPKY
GIRGIPTLLLFKNGEVAATKVGALSKGQLKEFLDANLAGSAGSAGSAGSAGSAGSAGSAGSAGSAGRSDK
IIHLTDDSFDTDVLKADGAILVDFWAEWSGPSKMIAPILDEIADEYQGKLTVAKLNIDQNPGTAPKYGIRGIP
TLLLFKNGEVAATKVGALSKGQLKEFLDANLAGSAGSAGSAGSAGSAGSAGSAGSAGSAGRSDKIIHLTD
DSFDTDVLKADGAILVDFWAEWSGPSKMIAPILDEIADEYQGKLTVAKLNIDQNPGTAPKYGIRGIPTLLLFK
NGEVAATKVGALSKGQLKEFLDANLAGSAGSAGSAGSAGSAGSAGSAGSAGSAGRSDKIIHLTDDSFDT
DVLKADGAILVDFWAEWSGPSKMIAPILDEIADEYQGKLTVAKLNIDQNPGTAPKYGIRGIPTLLLFKNGEVA
ATKVGALSKGQLKEFLDANLAGSAGSAGSAGSAGSAGSAGSAGSAGSAGRSDKIIHLTDDSFDTDVLKAD
GAILVDFWAEWSGPSKMIAPILDEIADEYQGKLTVAKLNIDQNPGTAPKYGIRGIPTLLLFKNGEVAATKVG
ALSKGQLKEFLDANLAGSAGSAGSAGSAGSAGSAGSAGSAGSAGRSDKIIHLTDDSFDTDVLKADGAILV
DFWAEWSGPSKMIAPILDEIADEYQGKLTVAKLNIDQNPGTAPKYGIRGIPTLLLFKNGEVAATKVGALSKG
QLKEFLDANLAGSAGSAGSAGSAGSAGSAGSAGSAGSAGRSDKIIHLTDDSFDTDVLKADGAILVDFWAE
WSGPSKMIAPILDEIADEYQGKLTVAKLNIDQNPGTAPKYGIRGIPTLLLFKNGEVAATKVGALSKGQLKEFL
DANLAGSAGSAGSAGSAGSAGSAGSAGSAGSAGRSDKIIHLTDDSFDTDVLKADGAILVDFWAEWSGPS
KMIAPILDEIADEYQGKLTVAKLNIDQNPGTAPKYGIRGIPTLLLFKNGEVAATKVGALSKGQLKEFLDANLA

Blue: Trx; **Yellow**: linker; **Pink**: N-terminal cysteine-glycine; White: restriction enzyme site.

**Nonamer (Trx-linker)$_4$(Trx-linker-24S/26C)(Trx-linker)$_4$**
SDKIIHLTDDSFDTDVLKADGAILVDFWAEWSGPSKMIAPILDEIADEYQGKLTVAKLNIDQNPGTAPKYGIR<br>GIPTLLLFKNGEVAATKVGALSKGQLKEFLDANLAGSAGSAGSAGSAGSAGSAGSAGSAGSAGRSDKIIHL<br>TDDSFDTDVLKADGAILVDFWAEWSGPSKMIAPILDEIADEYQGKLTVAKLNIDQNPGTAPKYGIRGIPTLLL<br>FKNGEVAATKVGALSKGQLKEFLDANLAGSAGSAGSAGSAGSAGSAGSAGSAGSAGRSDKIIHLTDDSF<br>DTDVLKADGAILVDFWAEWSGPSKMIAPILDEIADEYQGKLTVAKLNIDQNPGTAPKYGIRGIPTLLLFKNGE<br>VAATKVGALSKGQLKEFLDANLAGSAGSAGSAGSAGSAGSAGSAGSAGSAGRSDKIIHLTDDSFDTDVLK<br>ADGAILVDFWAEWSGPSKMIAPILDEIADEYQGKLTVAKLNIDQNPGTAPKYGIRGIPTLLLFKNGEVAATKV<br>GALSKGQLKEFLDANLAGSAGSAGSAGSAGSAGSAGSAGSAGSAGRSGTSDKIIHLTDDSFDTDVLKAD<br>GAILVDFWAEWSGPSKMIAPILDEIADEYQGKLTVAKLNIDQNPGTAPKYGIRGIPTLLLFKNGEVAATKVG<br>ALSKGQLKEFLDANLAGSAGSAGSAGSAGSAGSAGS**RRASAC**AGSAGSAGRSPRRSDKIIHLTDDSFDT<br>DVLKADGAILVDFWAEWSGPSKMIAPILDEIADEYQGKLTVAKLNIDQNPGTAPKYGIRGIPTLLLFKNGEVA<br>ATKVGALSKGQLKEFLDANLAGSAGSAGSAGSAGSAGSAGSAGSAGSAGRSDKIIHLTDDSFDTDVLKAD<br>GAILVDFWAEWSGPSKMIAPILDEIADEYQGKLTVAKLNIDQNPGTAPKYGIRGIPTLLLFKNGEVAATKVG<br>ALSKGQLKEFLDANLAGSAGSAGSAGSAGSAGSAGSAGSAGSAGRSDKIIHLTDDSFDTDVLKADGAILV<br>DFWAEWSGPSKMIAPILDEIADEYQGKLTVAKLNIDQNPGTAPKYGIRGIPTLLLFKNGEVAATKVGALSKG<br>QLKEFLDANLAGSAGSAGSAGSAGSAGSAGSAGSAGSAGRSDKIIHLTDDSFDTDVLKADGAILVDFWAE<br>WSGPSKMIAPILDEIADEYQGKLTVAKLNIDQNPGTAPKYGIRGIPTLLLFKNGEVAATKVGALSKGQLKEFL<br>DANLAGSAGSAGSAGSAGSAGSAGSAGSAGSAGRS
**Nonamer (Trx-linker)$_4$(Trx-linker-14S/16C)(Trx-linker)$_4$**
SDKIIHLTDDSFDTDVLKADGAILVDFWAEWSGPSKMIAPILDEIADEYQGKLTVAKLNIDQNPGTAPKYGIR<br>GIPTLLLFKNGEVAATKVGALSKGQLKEFLDANLAGSAGSAGSAGSAGSAGSAGSAGSAGSAGRSDKIIHL<br>TDDSFDTDVLKADGAILVDFWAEWSGPSKMIAPILDEIADEYQGKLTVAKLNIDQNPGTAPKYGIRGIPTLLL<br>FKNGEVAATKVGALSKGQLKEFLDANLAGSAGSAGSAGSAGSAGSAGSAGSAGSAGRSDKIIHLTDDSF<br>DTDVLKADGAILVDFWAEWSGPSKMIAPILDEIADEYQGKLTVAKLNIDQNPGTAPKYGIRGIPTLLLFKNGE<br>VAATKVGALSKGQLKEFLDANLAGSAGSAGSAGSAGSAGSAGSAGSAGSAGRSDKIIHLTDDSFDTDVLK<br>ADGAILVDFWAEWSGPSKMIAPILDEIADEYQGKLTVAKLNIDQNPGTAPKYGIRGIPTLLLFKNGEVAATKV<br>GALSKGQLKEFLDANLAGSAGSAGSAGSAGSAGSAGSAGSAGSAGRSGTSDKIIHLTDDSFDTDVLKAD<br>GAILVDFWAEWSGPSKMIVPILDEIADEYQGKLTVAKLNIDQNPGTAPKYGIRGIPTLLLFKNGEVAATKVG<br>ALSKGQLKEFLDANLAGSAGSAGSAG**RRASAC**SAGSAGSAGSAGSAGSAGRSPRRSDKIIHLTDDSFDT<br>DVLKADGAILVDFWAEWSGPSKMIAPILDEIADEYQGKLTVAKLNIDQNPGTAPKYGIRGIPTLLLFKNGEVA<br>ATKVGALSKGQLKEFLDANLAGSAGSAGSAGSAGSAGSAGSAGSAGSAGRSDKIIHLTDDSFDTDVLKAD<br>GAILVDFWAEWSGPSKMIAPILDEIADEYQGKLTVAKLNIDQNPGTAPKYGIRGIPTLLLFKNGEVAATKVG<br>ALSKGQLKEFLDANLAGSAGSAGSAGSAGSAGSAGSAGSAGSAGRSDKIIHLTDDSFDTDVLKADGAILV<br>DFWAEWSGPSKMIAPILDEIADEYQGKLTVAKLNIDQNPGTAPKYGIRGIPTLLLFKNGEVAATKVGALSKG<br>QLKEFLDANLAGSAGSAGSAGSAGSAGSAGSAGSAGSAGRSDKIIHLTDDSFDTDVLKADGAILVDFWAE<br>WSGPSKMIAPILDEIADEYQGKLTVAKLNIDQNPGTAPKYGIRGIPTLLLFKNGEVAATKVGALSKGQLKEFL<br>DANLAGSAGSAGSAGSAGSAGSAGSAGSAGSAGRS
**Blue**: Trx; **Yellow**: linker; **Green**: modified linker; **Pink**: sequence for modification; White: restriction enzyme site.

Table S2 Percentage residual currents ($I_{res\%}$) for the three levels of repeating feature A recorded during C-terminus first concatemer translocation.

	Trx-linker dimer	Trx-linker tetramer	Trx-linker hexamer	Trx-linker octamer	Trx-linker octamer[c]	Trx-linker octamer[d]
$I_{res\%}$ (A1)[a]	34 ± 1%	35 ± 1%	34 ± 1%	35 ± 1%	26 ± 2%	35 ± 1%<br>32 ± 1%
$I_{res\%}$ (A2)[a]	22 ± 3%	24 ± 2%	23 ± 1%	23 ± 1%	16 ± 1%	24 ± 1%
$I_{res\%}$ (A3)[a],[b]	1.9 ± 2.4%	1.7 ± 1.7%	2.2 ± 2.3%	2.3 ± 2.7%	0.5 ± 1.7%	2.0 ± 2.0%
N[a],[b]<br>n	105 units<br>2 separate pores	66 units<br>3 separate pores	122 units<br>1 pore	443 units<br>2 separate pores	65 units<br>3 separate pores	47 units<br>2 separate pores

[a] Ires% was calculated for each step in individual features A as the remaining current as a percentage of the open pore current (e.g., $I_{res\%}(A1) = I_{A1}/I_{open}\times100\%$). The standard deviations were derived for N Trx-linker units collected with ‘n’ separate pores. Conditions: 750 mM GdnHCl, 10 mM HEPES, pH 7.2, +140 mV (trans), 24 ± 1 °C. Concatemer concentrations (cis): dimer 2.23 μM, tetramer 0.63 μM, hexamer 0.25 μM, octamer 0.81 μM.

[b] Trx-linker units that produced a Level A3 with a dwell time <1 ms were discarded during analysis. The associated spikey appearance suggested under-sampling and therefore an inaccurate $I_{res\%}$ value. Trx-linker units that generated a Level A3 with a dwell time >1 ms and a square shape were included in the $I_{res\%}$ analysis.

[c] Conditions: 750 mM KCl, 10 mM HEPES, 0.81 μM octamer (cis), pH 7.2, +140 mV (trans), 24 ± 1 °C.

[d] Conditions: 750 mM GdnHCl, 10 mM HEPES, 0.81 μM octamer (trans), pH 7.2, -140 mV (trans), 24 ± 1 °C. A sub-conductance level was seen at Level A, which might be attributed to the folded thioredoxin unit adopting two conformations as a stopper under the electroosmotic force at the trans opening of the pore.

Table S3 Frequency of C terminus-first or N terminus-first translocation events recorded with Trx-linker concatemers[a].

	Voltage (trans)	C terminus-first translocation	N terminus-first translocation	N
Dimer	+140 mV	67%	33%	142
Tetramer	+140 mV	68%	32%	87
Hexamer	+140 mV	68%	32%	196
Octamer	+120 mV	86%	14%	87
	+140 mV	91%	9%	373
	+160 mV	94%	6%	192
	+180 mV	85%	15%	62

[a] Conditions: 750 mM GdnHCl, 10 mM HEPES, pH 7.2, the applied potential (trans) is specified in the table, 24 ± 1 °C. Concatemer concentrations (cis): dimer 2.23 μM, tetramer 0.63 μM, hexamer 0.25 μM, octamer 0.81 μM.

Table S4 Percentages of concatemer translocation events with N numbers of feature A repeats[a]

	N								Total events
	**1**	**2**	**3**	**4**	**5**	**6**	**7**	**8**	
Dimer[b]	44%	56%	/	/	/	/	/	/	73
Tetramer[b]	12%	31%	26%	31%	/	/	/	/	42
Hexamer[b]	14%	43%	20%	14%	3%	6%	/	/	35
Octamer[b]	10%	16%	22%	19%	3%	9%	9%	12%	86
Octamer[c]	11%	20%	29%	13%	9%	4%	7%	7%	45

[a] If the initial A3 level appeared as a spike to ~0 pA and was followed by at least one complete iteration of feature A, it was counted as one repeat.

[b] Conditions: 750 mM GdnHCl, 10 mM HEPES, pH 7.2, +140 mV (trans), 24 ± 1 °C. Concatemer concentrations (cis): dimer 2.23 μM, tetramer 0.63 μM, hexamer 0.25 μM, octamer 0.81 μM.

[c] Conditions: 750 mM KCl, 10 mM HEPES, pH 7.2, 0.81 μM octamer (cis), +140 mV (trans), 24 ± 1 °C.

Table S5 Mean dwell times ($<\tau>$) derived by QuB[a] for the three levels of repeating feature A (A1, A2, A3) recorded during the C-terminus first translocation of Trx-linker octamers through a single $(NN\_113R)_7$ nanopore[b]

Voltage (trans)	+140 mV	
**$<\tau_{A1}>$ / ms**	270 ± 20	N = 277
**$<\tau_{A2}>$ / ms**	23 ± 1	N = 277
**$<\tau_{A3}>$ / ms**	320 ± 60<br>0.69 ± 0.04	N = 40<br>N = 294

[a] Dwell time analysis was performed by using the maximum interval likelihood algorithm of QuB.
[b] Conditions: 750 mM GdnHCl, 10 mM HEPES, pH 7.2, 24 ± 1 °C.

Table S6 Percentage residual current ($I_{res\%}$) and root-mean-square noise (I_{RMS}) characteristics of individual modifications on Trx-linker nonamers.

	**$\Delta I_{res\%}$[a]**	**I_{RMS} / pA[c]**	**N**
**Trx-linker-14S-P**	4.4 ± 0.8%	0.96 ± 0.18	19 concatemers<br>4 separate pores
	4.0 ± 1.2%[b]	1.6 ± 0.9[b]	19 concatemers<br>4 separate pores
**Trx-linker-24S-P**	8.3 ± 1.6%	2.0 ± 0.6	27 concatemers<br>3 separate pores
	9.2 ± 2.1%[b]	2.5 ± 0.9[b]	23 concatemers<br>3 separate pores
**Trx-linker-16C-GSH**	5.1 ± 0.9%	0.93 ± 0.19	46 concatemers<br>4 separate pores
**Trx-linker-26C-GSH**	8.6 ± 1.3%	1.6 ± 0.2	21 concatemers<br>3 separate pores
**Trx-linker-16C-SLN**	15 ± 1%	0.73 ± 0.28	24 concatemers<br>3 separate pores
**Trx-linker-26C-SLN**	17 ± 2%	1.7 ± 0.5	55 concatemers<br>5 separate pores

[a] $\Delta I_{res\%}$ = <$I_{res\%}$(A1, Trx-linker)> – $I_{res\%}$(A1, Trx-linker+PTM). For a C terminus-first translocation event, <$I_{res\%}$(A1, Trx-linker)> was determined as the mean $I_{res\%}$ value of the unmodified A1 levels within an individual translocation event. $I_{res\%}$(A1, Trx-linker+PTM) was determined for the A1 level of the modified linker and appeared once per translocating concatamer. Conditions: 375 mM GdnHCl, 375 mM KCl, 10 mM HEPES, pH 7.2, +140 mV (trans), 24 ± 1 °C.

[b] Conditions: 750 mM GdnHCl, 10 mM HEPES, pH 7.2, +140 mV (trans), 24 ± 1 °C.

[c] Root-mean-square noise values (I_{RMS}) were measured from current traces after an applied post-recording filter at 2 kHz. I_{RMS} was normalised by the noise of each pore ($I_{RMS}^2 = I_{RMS}$(A1, Trx-linker+PTM)^2 - I_{RMS}(open pore)^2).

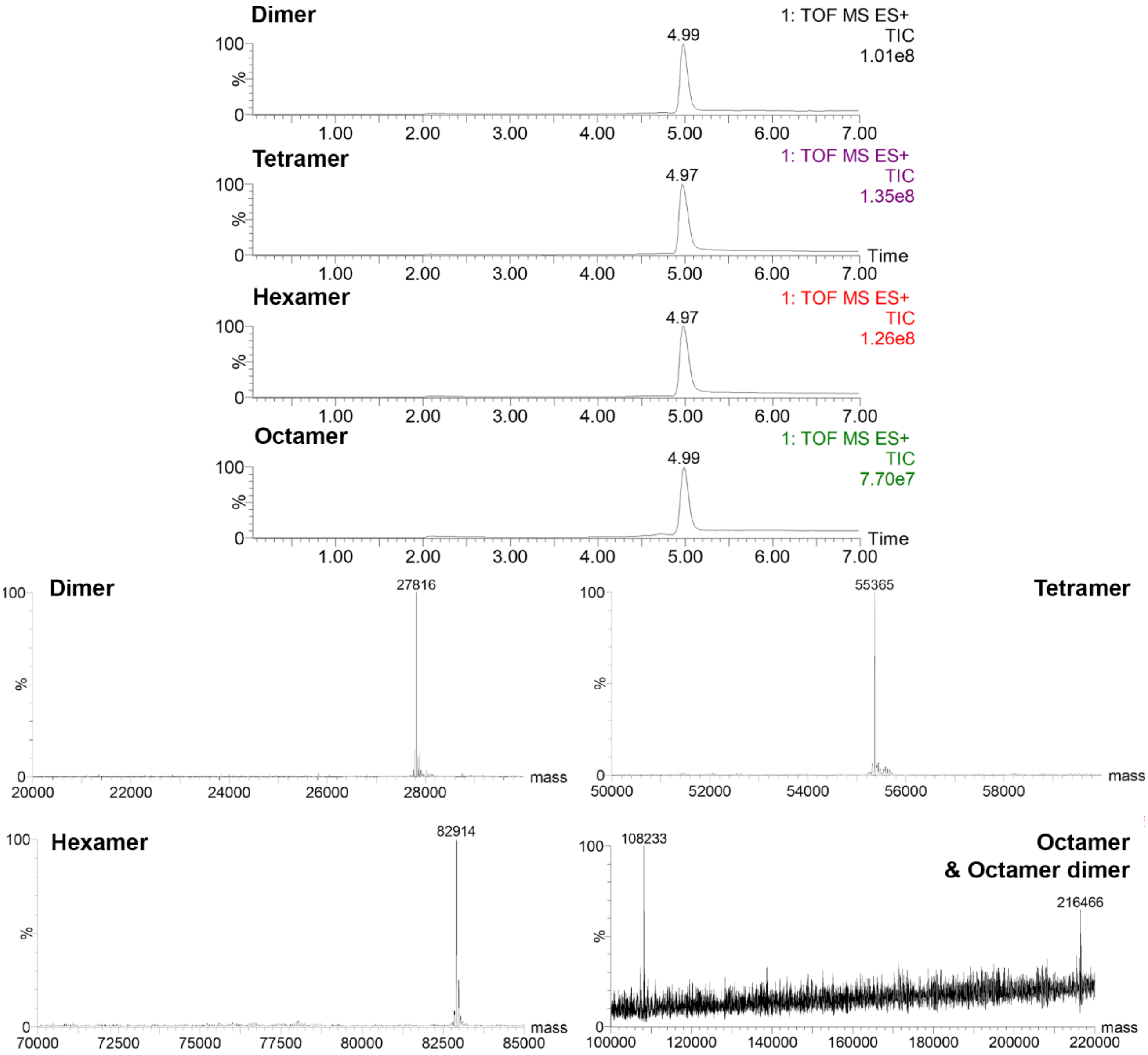

Fig. S1 LC-MS characterization of Trx-linker concatemers. LC-MS chromatograms (top) and deconvoluted ESI-MS spectra (bottom) are shown. Dimer, (Trx-linker)$_2$: mass = 27816 Da (calc) and 27816 Da (obs); Tetramer, (Trx-linker)$_4$: mass = 55367 Da (calc) and 55365 Da (obs); Hexamer, (Trx-linker)$_6$: mass = 82918 Da (calc) and 82914 Da (obs); Octamer, (Trx-linker)$_7$Trx: mass = 108231 Da (calc) and 108233 Da (obs); Dimer of octamers: mass = 216460 (calc) and 216466 Da (obs).

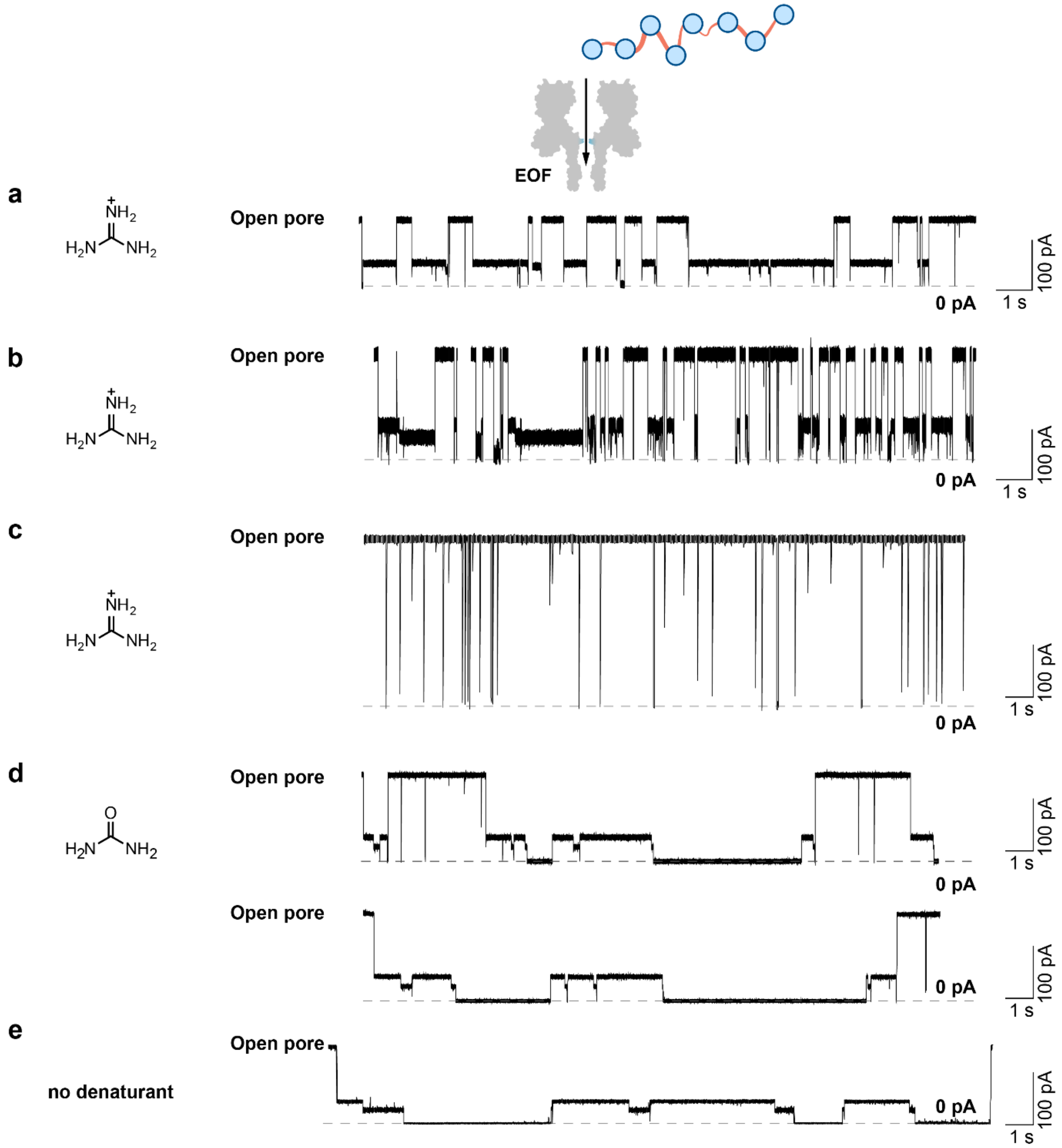

Fig. S2 Electroosmosis-driven translocation of Trx-linker octamers through a nanopore. a-e, Current traces for the translocation of Trx-linker octamers through a charge-selective nanopore ((NN-113R)$_7$) in the presence of 750 mM GdnHCl **(a)**, 1.5 M GdnHCl **(b)**, 3 M GdnHCl **(c)** without post-acquisition filtering, 2 M urea **(d)** or no denaturant **(e)** with 2 kHz post-acquisition filtering. Current features for subunit-by-subunit translocation were lost at 3 M GdnHCl **(c)**. The mean number of features A recorded per concatemer is **(a)** ~4, **(b)** ~3, **(c)** 0, **(d)** ~4, and **(e)** ~4. Conditions: 10 mM HEPES, pH 7.2, 0.81 μM Trx-linker octamer (cis), +140 mV (trans), 24 ± 1 °C, with **(a)** 750 mM GdnHCl; **(b)** 1.5 M GdnHCl; **(c)** 3 M GdnHCl; **(d)** 2 M urea and 750 mM KCl; **(e)** 750 mM KCl.

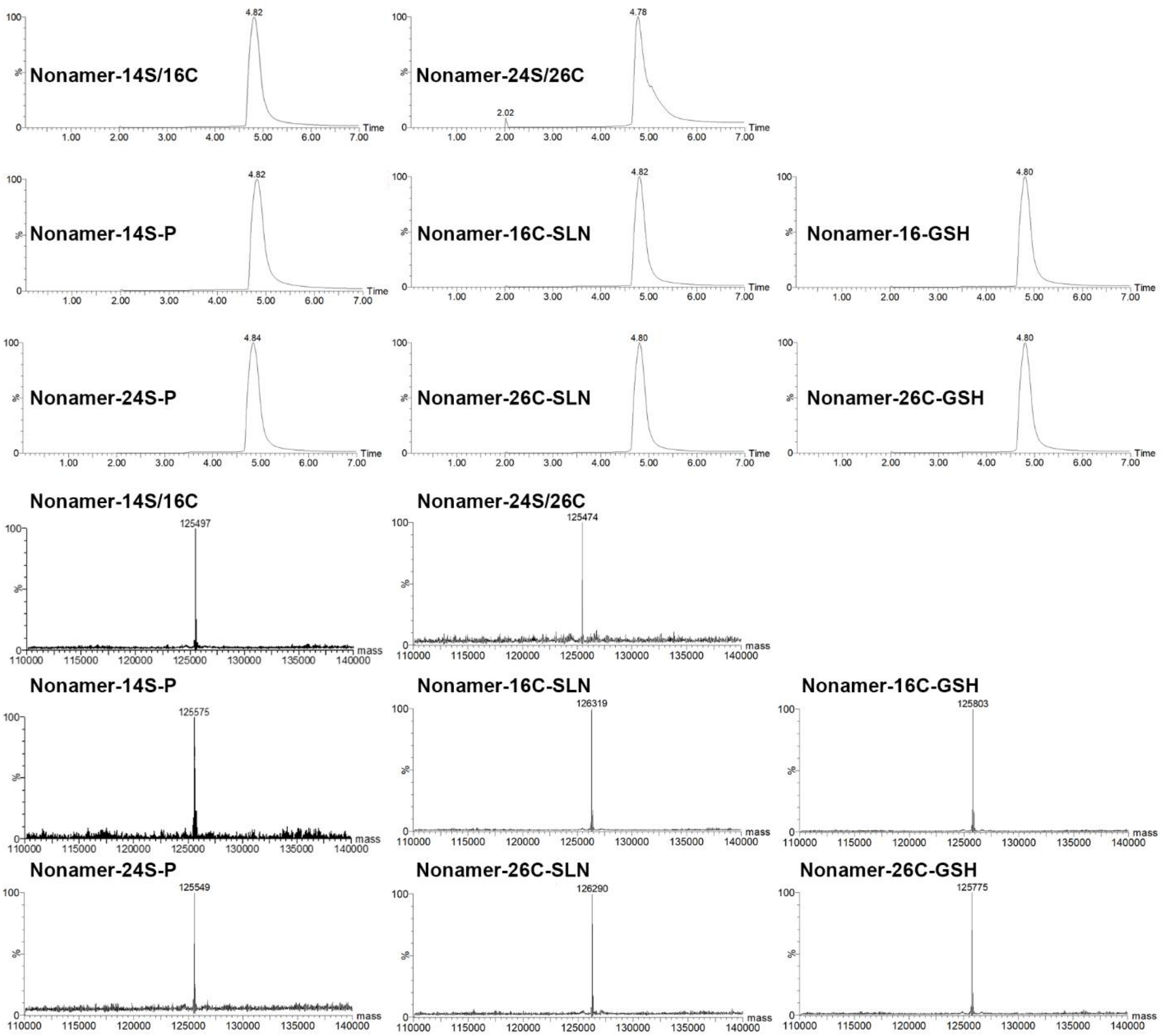

Fig. S3 LC-MS characterization of Trx-linker nonamers. LC-MS chromatograms (top) and deconvoluted ESI-MS spectra (bottom). Nonamer 14S/16C: mass = 125498 Da (calc) and 125497 Da (obs); Nonamer 24S/26C: mass = 125470 Da (calc) and 125474 Da (obs); Nonamer 14S-P: mass = 125578 Da (calc) and 125575 Da (obs); Nonamer 16C-SLN: mass = 126319 Da (calc) and 126319 Da (obs); Nonamer 16C-GSH: mass = 125803 (calc) and 125803 Da (obs); Nonamer 24S-P: mass = 125550 Da (calc) and 125549 Da (obs); Nonamer 26C-SLN: mass = 126291 Da (calc) and 126290 Da (obs); Nonamer 26C-GSH: mass = 125775 (calc) and 125775 Da (obs).

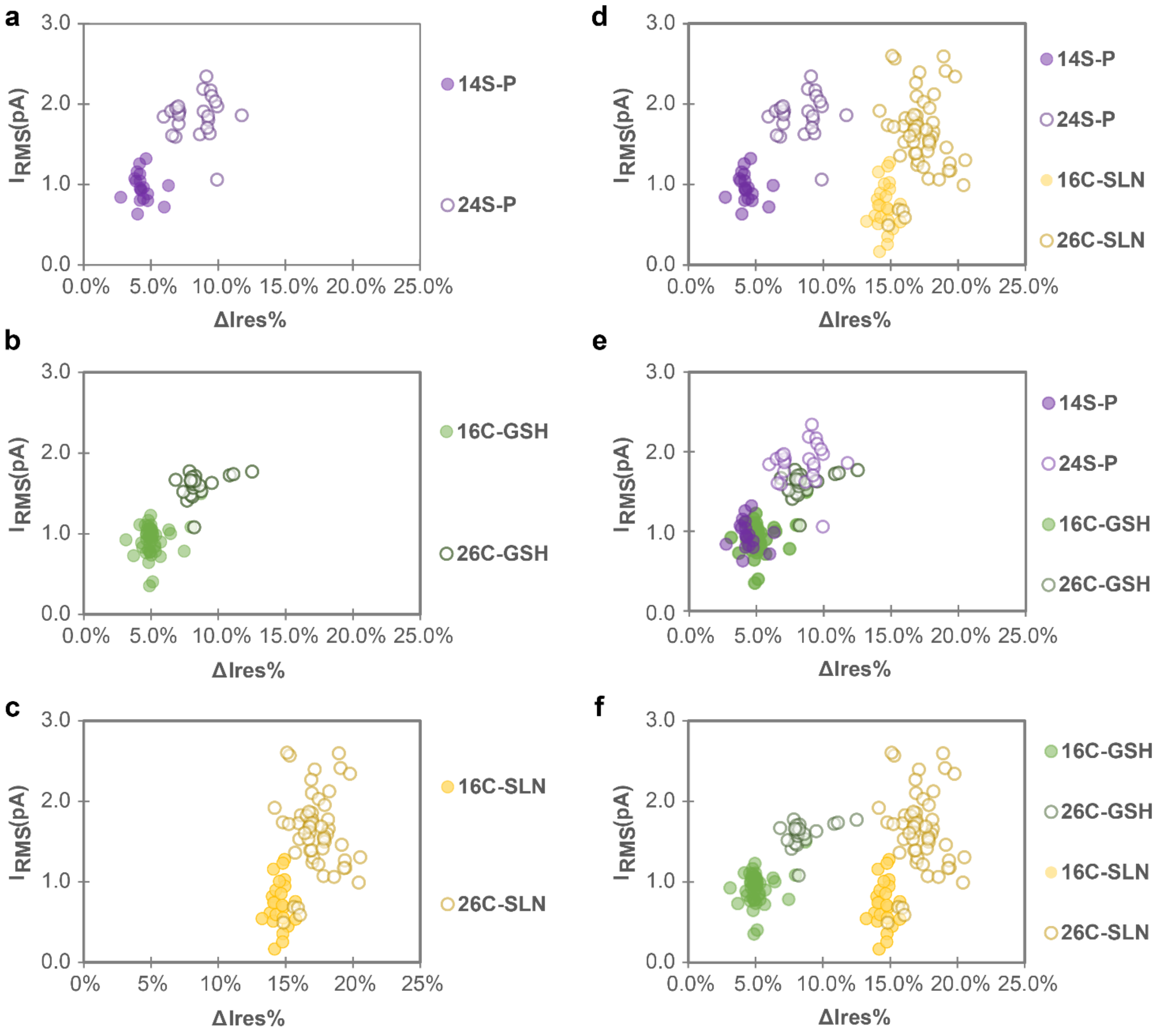

Fig. S4 Identification and positional discrimination of PTMs in protein concatemers translocated by electroosmotic flow through a nanopore. Protein nonamers containing a single PTM (See Fig. 3 for protein sequences and PTM structures) were tested. **a-c**, Scatter plots of I_{RMS} and $\Delta I_{res\%}$ showing positional discrimination of a phosphorylated serine, a glutathionylated cysteine, or a glycosylated cysteine at sites 10 aa apart ($\Delta I_{res\%}$ = <$I_{res\%}$(A1, Trx-linker)> – $I_{res\%}$(A1, Trx-linker+PTM), where <$I_{res\%}$(A1, Trx-linker)> is the mean $I_{res\%}$ value of A1 levels of an unmodified unit within a single translocation event. Conditions: 375 mM GdnHCl, 375 mM KCl, 10 mM HEPES, pH 7.2, 1.2 μM Trx-linker nonamer (cis), +140 mV (trans), 24 ± 1 °C. **d-f,** Overlaid scatter plots of I_{RMS} and $\Delta I_{res\%}$ showing discrimination between phosphorylated and glycosylated populations, glutathionylated and glycosylated populations, and overlaps between phosphorylated and glutathionylated populations.

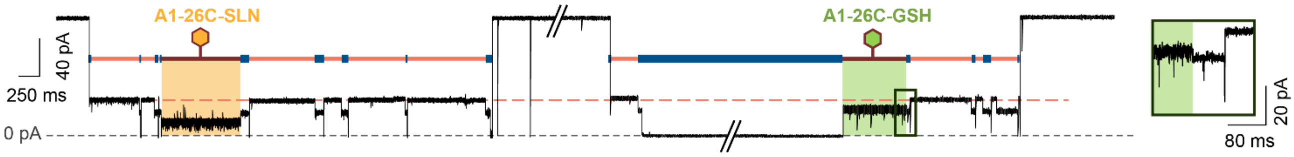

Fig. S5 Identification of PTMs in a mixture of two concatemers. Current traces recorded with the same nanopore for three C terminus-first translocations of a mixture of two Trx-linker nonamers containing either a GSH or SLN modification at position 26C within the central linker. The A1 level for the unmodified units (orange dash), and the A1 level for a unit modified with 26C-GSH (A1-26C-GSH, green) or 26-SLN (A1-26C-SLN, yellow) are colour-coded. The inset zooms in on the transition from A1-26C-GSH to A2. Traces have been filtered at 2 kHz. Conditions: 375 mM GdnHCl, 375 mM KCl, 10 mM HEPES, pH 7.2, 1.2 μM Trx-linker nonamer (cis), +140 mV (trans), 24 ± 1 °C.

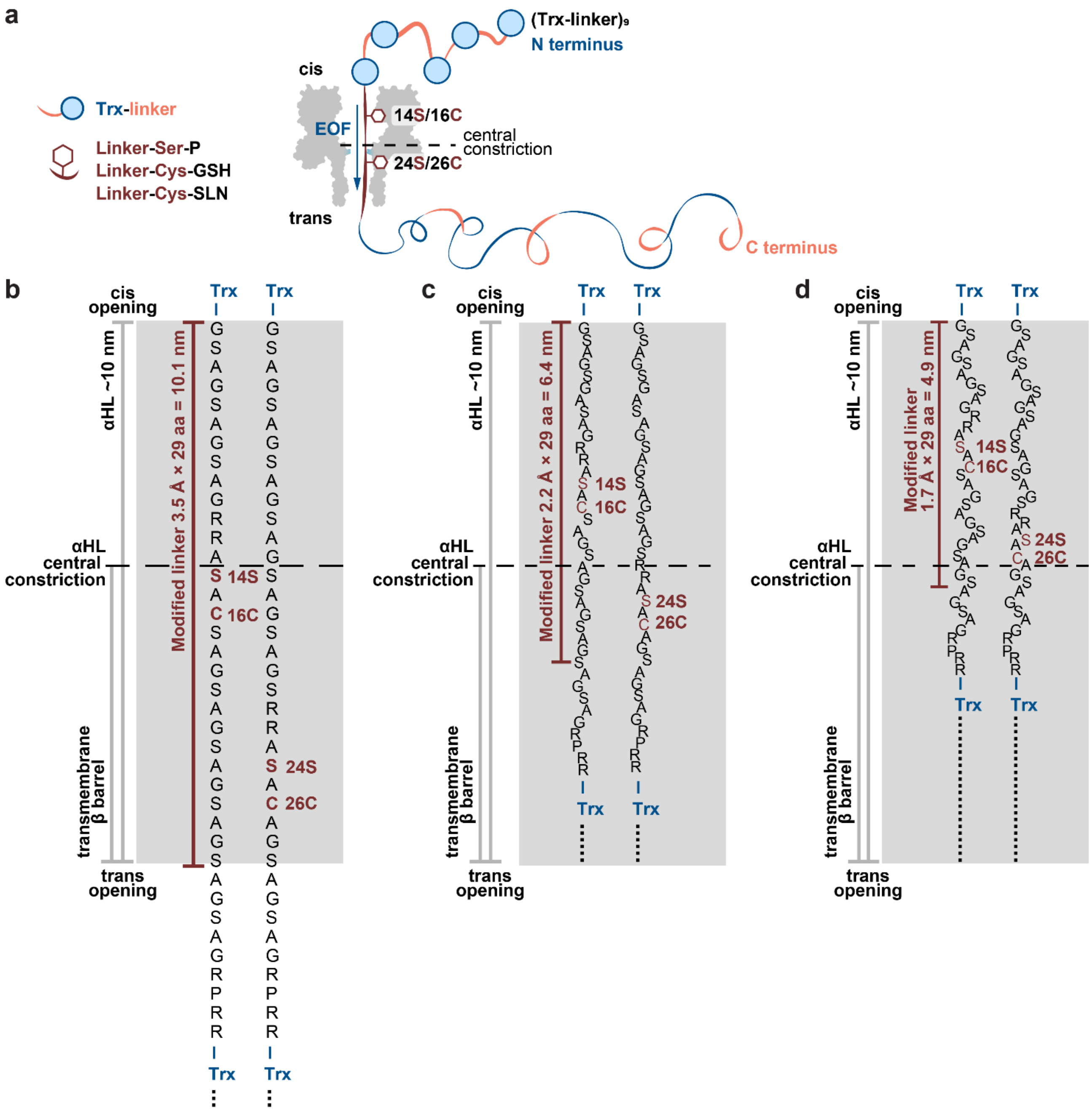

Fig. S6 Positions of modification sites during translocation through an αHL pore. a, The Trx-linker nonamers contained a RRASAC sequence within the central linker, which was post-translationally modified (hexagon). In a C-terminus-first threading configuration, as shown, the 14S/16C modification sites would be located closer to the cis opening of the αHL pore than the 24S/26C pair, when translocation is paused with a Trx unit at the cis mouth of the pore. **b-d,** Depending on the degree of extension of the polypeptide chain under the EOF (3.5 Å per aa when fully extended, 1.7-2.2 Å per aa under ~5-10 pN[1]), the 14S/16C and 24S/26C sites would be located at different positions within an αHL pore. Assuming that the N-terminal residue of the linker is at the cis opening of the pore when the translocation is arrested by a folded Trx unit, the modified linker (red) might fully span the αHL pore (**b**) or occupy only a part of the nanopore (**c,d**). When the 24S/26C sites are located nearer the central constriction of the αHL pore (**c,d**), a PTM at 24S/26C would produce a larger current blockade than that at 14S/16C (PTM = Ser-P, Cys-GSH, Cys-SLN), which is what is observed (Fig. 3b). Given that the applied potential drops mostly across the transmembrane β barrel[2], the current difference between 14S/16C+PTM and 24S/26C+PTM is likely to be larger in **c** than in **d**.

References

1. Winardhi, R. S., Tang, Q., Chen, J., Yao, M. & Yan, J. Probing small molecule binding to unfolded polyprotein based on its elasticity and refolding. *Biophys. J.* **111**, 2349–2357 (2016).
2. Howorka, S. & Bayley, H. Probing distance and electrical potential within a protein pore with tethered DNA. *Biophys. J.* **83**, 3202–3210 (2002).
