# Supplementary figures and images for: Enzyme-less nanopore detection of post-translational modifications within long polypeptides
Source: Nat Nanotechnol. 2023 Jul 27;18(11):1335–40. doi: 10.1038/s41565-023-01462-8 (PMC10656283; doi:10.1038/s41565-023-01462-8)

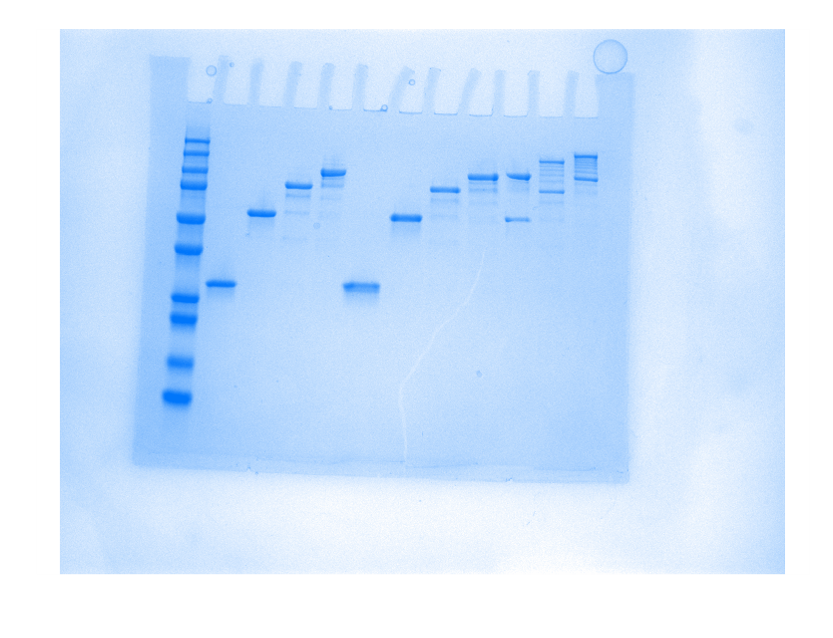

Supplement: Supplementary file 2 — Unprocessed sodium dodecyl sulfate–polyacrylamide gel. [file 41565_2023_1462_MOESM2_ESM.tif]
